# Altered brain structural and functional connectivity in cannabis users

**DOI:** 10.1038/s41598-023-32521-8

**Published:** 2023-04-10

**Authors:** Najme Soleimani, Kamran Kazemi, Mohammad Sadegh Helfroush, Ardalan Aarabi

**Affiliations:** 1 Department of Electrical Engineering, Shiraz University of Technology, Shiraz, Iran; 2 Faculty of Medicine, University of Picardie Jules Verne, Amiens, France; 3 Laboratory of Functional Neuroscience and Pathologies, University Research Center, University Hospital, Amiens, France

**Keywords:** Network models, Computational models

## Abstract

Cannabis is one of the most used and commodified illicit substances worldwide, especially among young adults. The neurobiology mechanism of cannabis is yet to be identified particularly in youth. The purpose of this study was to concurrently measure alterations in brain structural and functional connectivity in cannabis users using resting-state functional magnetic resonance images (rs-fMRI) and diffusion-weighted images (DWI) from a group of 73 cannabis users (age 22–36, 19 female) in comparison with 73 healthy controls (age 22–36, 14 female) from Human Connectome Project (HCP). Several significant differences were observed in local structural/functional network measures (e.g. degree and clustering coefficient), being prominent in the insular and frontal opercular cortex and lateral/medial temporal cortex. The rich-club organization of structural networks revealed a normal trend, distributed within bilateral frontal, temporal and occipital regions. However, minor differences were found between the two groups in the superior and inferior temporal gyri. Functional rich-club nodes were mostly located within parietal and posterior areas, with minor differences between the groups found mainly in the centro-temporal and parietal regions. Regional network measures of structural/functional networks were associated with times used cannabis (TUC) in several regions. Although the structural/functional network in both groups showed small-world property, no differences between cannabis users and healthy controls were found regarding the global network measures, showing no association with cannabis use. After FDR correction, all of the significant associations between network measures and TUC were found to be insignificant, except for the association between degree and TUC within the presubiculum region. To recap, our findings revealed alterations in local topological properties of structural and functional networks in cannabis users, although their global brain network organization remained intact.

## Introduction

Cannabis is one of the most commonly used illicit drugs worldwide, and its consumption has been on the rise in recent years, coinciding with its legalization in many countries^1^. Research has shown that dependence on cannabis is associated with a range of neurocognitive deficits, including impaired episodic memory^2^, engagement in risky behaviors, and poor performance on cognitive tasks that require executive function^1,3^.

In the past decades, morphometry and network analyses have been commonly used in most studies to investigate the association between cannabis use and brain structure and function. The morphometry based approach is used to study changes in the local concentration (volume/thickness) of brain tissues^4^. Early studies found no significant morphological alterations in the brain associated with chronic cannabis use^5^. However, recent studies have shown that the use of cannabis may lead to hippocampal, parahippocampal and lateral atrophy^6–9^. Alterations in brain function and structure may not be merely due to local changes in brain morphology, it can be also a result of changes in interactions between brain regions.

Through modeling the brain as a network^10^, several studies have explored alterations in brain functional and structural connectivity attributed to chronic cannabis use using resting-state functional and diffusion weighted imaging data^11–14^. Previous studies on large-scale brain networks have reported heterogeneous findings regarding the association of cannabis with brain structural and functional connectivity patterns in cannabis users. Initial findings suggested less efficient structural brain networks in addition to altered local structural connectivity in the cingulate region in a group of cannabis users using graph theoretical measures^12^. One of the first studies examining the impact of long-term cannabis use on axonal connectivity found impaired structural connectivity in the splenium of the corpus callosum, fornix and commissural fibers^15^. Regular cannabis users were found to have increased structural fractional anisotropy, which then decreased alongside heavy use^3^. Other studies^16–18^ have found no significant differences in the global properties of brain structural networks between cannabis users and controls.

Long-term cannabis use is shown to be associated with a wide variety of changes in functional connectivity, although most studies focused on distinct brain regions using cognitive tasks^19,20^. A few studies investigated resting-state functional connectivity across large-scale brain networks^16,21^. Manza et al.^11^ found increased local functional connectivity in the ventral striatum, midbrain, brainstem and lateral thalamus. Whilst, using seed-based connectivity analysis, they reported no significant differences in whole-brain functional connectivity between cannabis users and healthy controls using the aforementioned regions as seed. Ramaekers et al.^22^ found a broad state of hyperconnectivity within the major brain networks such as dorsal attention, limbic, subcortical and cerebellum network in chronic cannabis users in comparison with acute users. Using the graph theoretical analysis, no differences in global and regional properties of resting-state functional networks were found between cannabis users and non-users^21^.

In recent years, there has been growing interest in identifying densely-connected hubs (so-called “rich club”) in brain networks, shown to play a critical role in information integration across structural and functional brain networks^23,24^. Few studies have examined the rich club organization of structural brain networks in cannabis users in comparison with non-users^16,17^. Despite the bulk of studies, alterations in both functional and structural connectivity of brain networks have not been fully explored in cannabis users.

In the present study, we aimed at exploring alterations in brain functional and structural connectivity and the rich club organization of structural and functional brain networks using graph theoretical metrics in cannabis users in comparison with healthy controls. We further assessed the association between times used cannabis and network measures.

## Materials and methods

### Subjects

This study included 146 subjects from the HCP dataset (final release, https://www.humanconnectome.org/study/hcp-young-adult/document/1200-subjects-data-release)^25^. All the participants provided *written informed* consents. From this cohort (n = 1206; aged 22–36; 54% female), 109 individuals met the DSM-IV criteria for cannabis dependence and had both rs-fMRI and DWI imaging data. From this subgroup, individuals with comorbid alcohol dependence, outliers on DSM levels of anxiety and depression (> 3 SD from the mean of all 1206 HCP subjects) in addition to those individuals with low quality outlier images were excluded^19^. The final sample included 73 cannabis users. Since it is recommended to match groups based on demographic and lifestyle factors, a critical step to minimize the potential confounding effects of these factors^11^, a group of 73 healthy control group was selected from 1096 healthy subjects included in the HCP, well matched with the cannabis group on age, sex, education, BMI, alcohol and tobacco usage^11^ using the MatchIt function in R with (*p* > *0.1*). Subjects’ socio-demographic information is provided in Table 1.

**Table 1:** Summary of socio-demographic and substance use characteristics of the subjects included in the study.

		Cannabis users	Healthy controls	p-value	t-statistic	df
N of total		73	73			
Mean Age^a^ (SD)		28.58 (3.69)	27.72 (3.56)	0.1352	1.51	72
Gender (N of Male (%))		54 (73.97%)	59 (80.82%)			
Mean BMI (SD)		26.99 (4.91)	27.06 (4.54)	0.9309	− 0.087	72
Education (Years of education completed)	< 11	6 (8.21%)	3 (4.10%)	0.8163	− 0.233	72
	12	9 (12.32%)	15 (20.54%)			
	13	11 (15.06%)	4 (5.47%)			
	14	10 (13.69%)	13 (17.8%)			
	15	6 (8.21%)	5 (6.84%)			
	16	22 (30.13%)	25 (34.24%)			
	17 +	9 (12.32%)	8 (10.95%)			
Times used Cannabis (lifetime)	0 (never used)	–	41 (56.16%)	2.2014e−43	31	72
	1 (1–5 times)	–	23 (31.5%)			
	2 (6–10 times)	–	9 (12.32%)			
	3 (11–100 times)	13 (17.8%)	–			
	4(101–999 times)	20 (27.39%)	–			
	5 (> 1000 times)	40 (54.79%)	–			
Age at first use of cannabis	1 (< = 14)	23 (31.5%)	–			
	2 (15–17)	32 (43.83%)	–			
	3 (18–20)	15 (20.54%)	–			
	4 (> = 21)	3 (4.10%)	–			
Mean Alcohol use (SD)		0.31 (0.51)	0.32 (0.55)	0.9489	− 0.064	72
Mean Tobacco use (SD)		0.24 (0.78)	0.15 (0.59)	0.4566	0.748	72

^a^Age range = 22–36 years.

### Neuroimaging data

From each subject, imaging data were acquired on a Siemens 3T scanner with a 32-channel coil at Washington University as described in^26^. The 3D T1- and T2- weighted MR images were acquired at 0.7 mm isotropic resolution (FOV = 224 mm, matrix = 320, 256 slices). The diffusion-weighted images (DWI) were acquired with a high spatial resolution of 1.25 mm isotropic (TR/TE = 5520 ms/89.5 ms) using a high-angular resolution diffusion imaging (HARDI) approach, including six runs and three different shells of b = 1000, 2000 and 3000 s/mm2 with 270 q-points distributed over the three shells. The rs-fMRI data were acquired in two sessions and two runs of approximately 15 min each (one LR and another RL phase encoding) in each session with an EPI sequence (Multiband factor = 8, TR/TE = 720 ms/33.1 ms, flip angle = 52°, FOV = 208 mm, spatial resolution = 2 × 2 × 2 mm). For rs-fMRI, participants were instructed to lie with eyes open, to relax and look at a white cross on a dark background, think of nothing and not to fall asleep.

#### Data preprocessing

The T1w images were minimally preprocessed for spatial distortion and motion correction, and normalization in the MNI space^27^. The diffusion weighted images were also preprocessed for b0 intensity normalization, EPI distortion correction, eddy current and motion correction, and gradient nonlinearity correction^28^. We used all rs-fMRI data in the “CIFTI” format, that is, combinations of cortical gray matter data modeled on surfaces and subcortical gray matter data modeled in volumetric parcels included in one image. All functional images were minimally preprocessed for gradient unwarping, EPI distortion correction, motion correction, registration to T1w scans, high pass filtering with a cut-off of 2000 s used for linear detrending, ICA-based denoising in order to automatically remove artifactual, bad and very low-frequency components, and non-linear normalization to MNI space, described in detail elsewhere^28^. In the HCP pre-processing pipeline, the independent component analysis (MELODIC, FSL-FIX) was used to remove artifactual and "bad" components, as well as non-neural spatiotemporal components from 15-min high-pass filtered rs-fMRI data. To avoid removing variance of interest from the data, a conservative non-aggressive approach was further used with a cut-off of 2000 s shown to be more appropriate than 200 s for ICA-FIX^29^. The rs-fMRI images were also cross-registered across subjects using “MSMall” algorithm^30^, which aligns functional networks using features derived from myelin, resting state networks, and rs-fMRI visuotopic maps for better registration of functional cortical areas in comparison with legacy pipelines^30,31^*.*

### Tractography

The tractography for each individual was performed using the deterministic generalized Q-sampling imaging (GQI) fiber tracking algorithm^32^ with DSI Studio (http://dsi-studio.labsolver.org/). The optimal value for diffusion sampling length ratio was set to 1.25 to better model cross fibers in regions like the lateral corpus callosum. For each subject, 1,000,000 fibers were generated and quantitative anisotropy (QA) was computed for the orientation distribution function (ODF) in each voxel^33^. The QA is a robust index with less sensitivity to the partial volume effect of crossing fibers besides higher resolution compared to FA-aided tractography^33^. Streamlines shorter than 30 mm and longer than 300 mm were discarded as suggested in^34^. Finally, the topology-informed pruning algorithm^35^ was applied to remove false positive connections.

### Network construction

To construct functional and structural brain graphs, we used the Glasser atlas^30^ containing 360 regions (180 areas per hemisphere). Since subcortical regions have been commonly included in addiction studies^11^, we used the modified version of this atlas of 379 parcels including 19 subcortical regions. The parcellation scheme was based on alterations in cortical architecture, function, connectivity and topography of the brain in 210 young healthy adults from HCP^30^.

For each participant, a structural connectivity matrix with N × N elements representing the normalized QA between regions was constructed. The optimal threshold was set to 0.1% of the maximum value (default threshold in DSI Studio) of structural connectivity for each individual. A weighted group structural matrix was then computed for each group by averaging the connectivity matrix elements for connections present in at least 75% of the subjects^23^.

**Figure 1:**
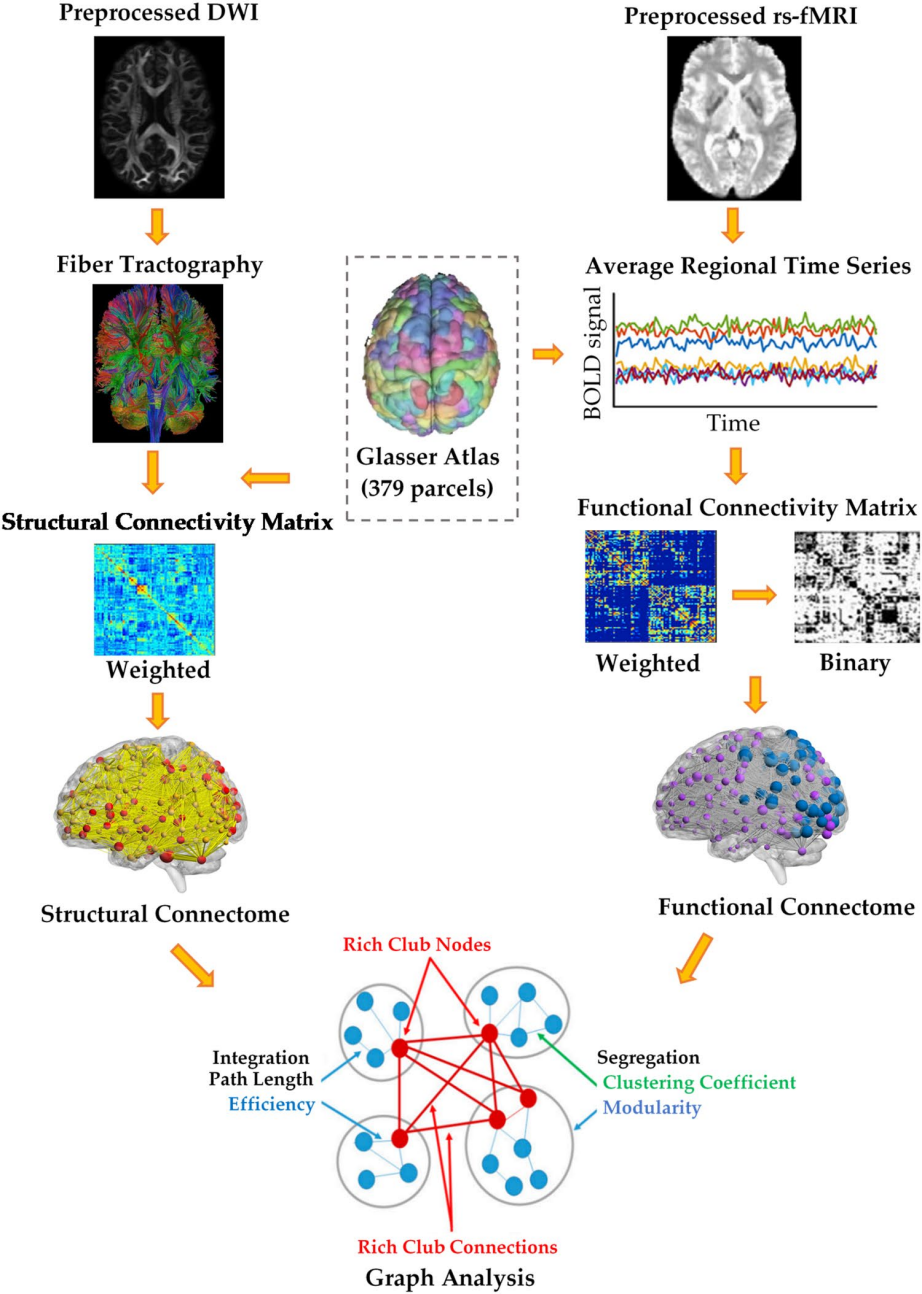
Processing pipeline for brain structural and functional Network Analysis. A structural connectome was constructed for each individual using fiber tractography and a parcellation scheme. A functional connectome was also constructed for each individual by calculating the pairwise Pearson's correlation coefficient of the average time courses of 379 regions. A graph-theoretical analysis was then performed to investigate the topological properties and rich-club organization of the structural and functional brain networks in both healthy controls and cannabis users.

Furthermore, a functional connectivity matrix was constructed for each individual by calculating the pairwise Pearson's correlation coefficient of the average time courses of 379 regions. The functional connectivity matrices were then thresholded^27^ using an optimal threshold of 0.2, preserving 20% of the strongest connections. The optimal threshold was obtained based on the trade-off between density and global efficiency^36^. A binary group functional matrix was also computed for both groups by averaging the individual matrices by preserving 20% of the strongest connections. The whole procedure is depicted in Fig. 1.

#### Network topological properties

To explore the association of cannabis use with brain structural and functional connectivity, the topological properties of both the structural and functional networks were computed at the single subject and group levels using the Brain Connectivity Toolbox (BCT, http://www.brain-connectivity-toolbox.net/). To characterize the brain network topology, metrics of network integration (characteristic path length, global efficiency and degree), segregation (clustering coefficient and modularity) as well as small-worldness were computed for each network. Detailed information about each property can be found in^37^ and^10^.

#### Rich-Club organization

We further investigated the effect of cannabis on the rich-club organization of the brain using the method described in^23,24^. To this end, an unweighted rich-club coefficient was computed for the average functional network of each group. For each k in the range of [1, maximum degree in network] the rich club coefficient ϕ(k) was computed as the ratio of the number of connections in the subgraph defined by nodes with degree less than k, relative to the total number of possible connections in the subgraph.

1 $$\phi \left(k\right)=\frac{2{E}_{k}}{{N}_{k}\left({N}_{k}-1\right)}$$

where ${E}_{k}$ is the number of connections with a degree less than k, and ${N}_{k}\left({N}_{k}-1\right)$ is the total number of possible connections.

Following a similar procedure, a weighted rich-club coefficient ${\phi }_{k}^{w}$ was computed for each group structural network. After ranking all weights of the structural network (${w}^{ranked}$), ${\phi }^{w}\left(k\right)$ was computed as follows:

2 $${\phi }^{w}\left(k\right)=\frac{{w}_{k}}{{\sum }_{l}^{{E}_{k}}{w}_{l}^{ranked}}$$

where ${w}_{k}$ is the sum of weights on the links in the subgraph of nodes with a rank greater than k, and ${w}^{ranked}$ is a vector of all link weights in the structural network, ranked from largest to smallest weight.

The normalized rich-club coefficient ϕ_norm_(k) of structural and functional networks for each group were then computed with respect to ϕ_random_(k), which was computed as the average rich-club coefficient over 1000 random networks of equal size and similar connectivity distribution^23^ to test whether the rich club of the actual network significantly exceeded that of a null model or not by with *p* < *0.05*. For the structural and functional networks of cannabis users and healthy controls, ϕ_norm_(k) greater than 1 within a range of k with *p* < *0.05* revealed the presence of rich-club nodes. In the present study, we chose the k level in a way that 30% of the network nodes identified as rich-club nodes.

### Statistical analysis

Differences in global and local graph metrics between cannabis users and healthy controls were assessed via t-test. Furthermore, a linear regression analysis at the nodal level was used to examine the relationship between the structural/functional network measures (degree and clustering coefficient) and times used cannabis (TUC) in cannabis users. We presented our findings using a range of statistical significance thresholds (*p* < *0.05, p* < *0.02, p* < *0.01, and p* < *0.005*), both uncorrected and corrected for multiple comparisons using the false discovery rate (FDR), mainly because corrections for multiple comparisons can be overly conservative when dealing with a large number of nodes.

### Informed consent

Informed consent was obtained from all subjects involved in the study.

## Results

### Graph measures

As reported in Table 2, no significant differences (*p* > *0.05*) were found in global network measures (global efficiency, characteristic path length, modularity and small-worldness) for either structural or functional networks between cannabis users and healthy controls.

**Table 2:** Average values (mean ± SD) of structural and functional network properties for each group.

Topological characteristic	Structural network					Functional network				
	Cannabis users	Healthy controls	p-value	t-statistic	df	Cannabis users	Healthy controls	p-value	t-statistic	df
Global efficiency	0.3185 ± 0.0215	0.3184 ± 0.0223	0.99	− 0.007	144	0.4864 ± 0.0292	0.4938 ± 0.0265	0.11	1.60	144
Characteristic path length	0.8234 ± 0.0652	0.8176 ± 0.0650	0.58	− 0.54	144	1.9604 ± 0.055	1.9538 ± 0.047	0.43	− 0.77	144
Modularity	0.3308 ± 0.0227	0.3222 ± 0.0280	0.05	− 2.03	144	0.2616 ± 0.0530	0.2697 ± 0.0459	0.33	0.97	144
Small-worldness	1.5792 ± 0.0928	1.5563 ± 0.1092	0.17	− 1.35	144	1.3042 ± 0.1939	1.3483 ± 0.1916	0.16	1.40	144
Degree	77.76 ± 5.21	78.53 ± 5.54	0.38	0.86	144	75.59 ± 1.43	75.59 ± 1.43	1	0	144
Clustering coefficient	0.2862 ± 0.02	0.2855 ± 0.0207	0.83	− 0.20	144	0.6257 ± 0.0183	0.6265 ± 0.0188	0.79	0.25	144

**Figure 2:**
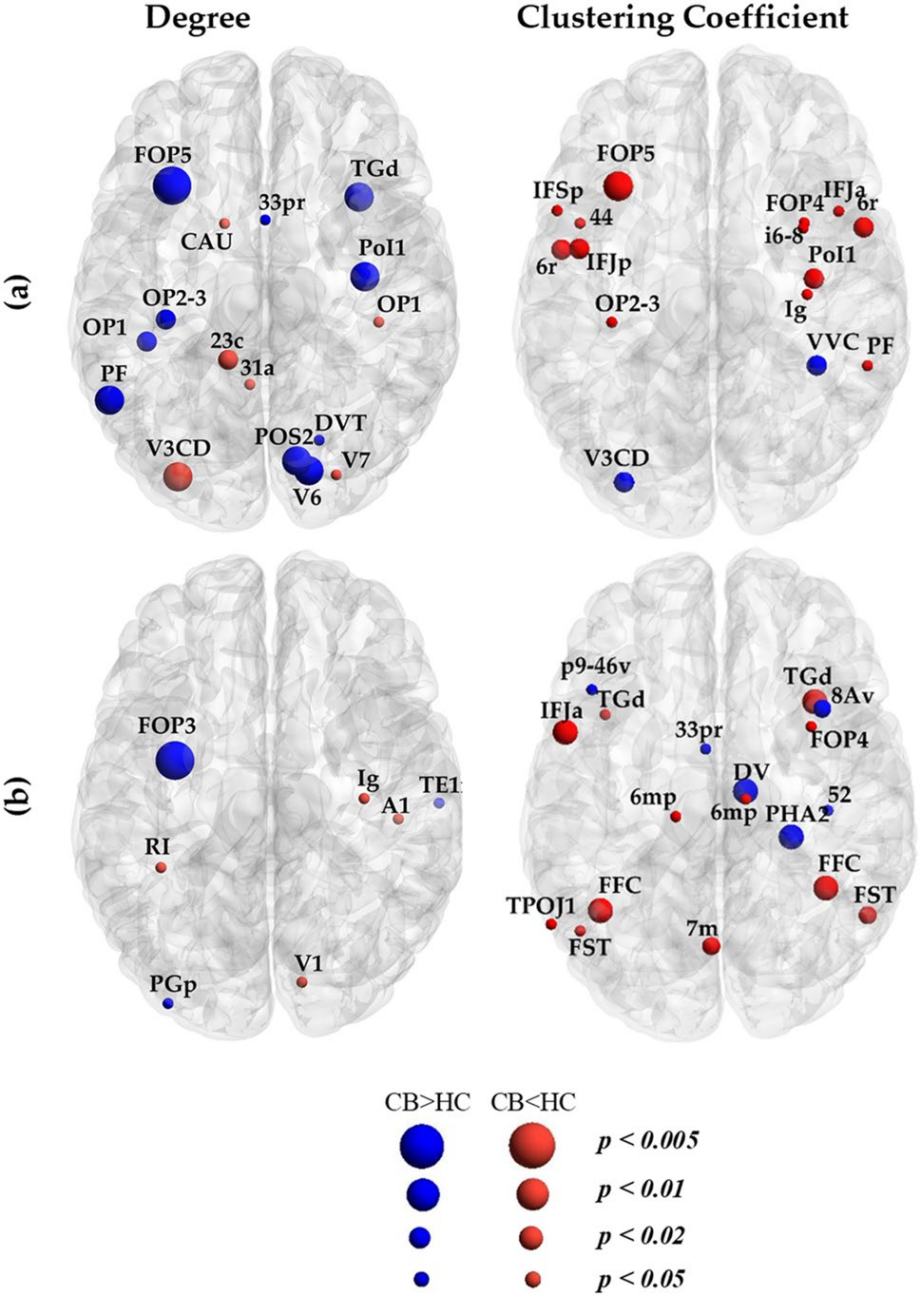
Regions showing differences in degree and clustering coefficient between cannabis users and healthy controls in (**a**) structural networks and (**b**) functional networks. The color of nodes indicates significant increases (red) or decreases (blue) in degree and clustering coefficient for cannabis users (CB) compared to healthy controls (HC). The size of nodes represents between group differences with *p* < *0.05, p* < *0.02*, *p* < *0.01* and *p* < *0.005* (*uncorrected*) with larger nodes showing smaller p values.

Figure 2 and Tables S1–S4 show significant differences (with *p* < *0.05*, *p* < *0.02*, *p* < 0.01 and *p* < *0.005*, *uncorrected*) in nodal degree and clustering coefficient for structural and functional networks between the groups. As illustrated, the structural networks in cannabis users compared to controls displayed lower (*p* < *0.01, uncorrected*) degree centrality within the left frontal opercular, posterior opercular cortex, inferior parietal cortex, and in right lateral temporal, posterior cingulate and visual areas. Few nodes in the left parieto-occipital regions including V3CD showed increased structural degree in cannabis users relative to controls. In the functional networks, the left frontal operculum showed significant decreases (*p> 0.005, uncorrected*) in degree in cannabis users.

Cannabis users further exhibited higher local segregation (clustering coefficient, *p< 0.01 uncorrected*) within the frontoparietal regions including the premotor cortex, frontal opercular and inferior frontal cortices for structural networks. Few regions in posterior areas including the ventral stream visual cortex and V3CD showed lower clustering coefficients in cannabis users.

The functional networks in cannabis users were also characterized by increased clustering coefficient in the left inferior frontal cortex, ventral stream visual cortex, FST, and area TG dorsal. Compared to controls, the cannabis group exhibited lower local functional segregation within the right hemisphere in the dorsolateral prefrontal cortex, ParaHippocampal area 2 and Diencephalon ventral area.

Overall, none of the aforementioned significant differences between the cannabis users and healthy controls survived after FDR correction.

### Rich-Club organization of structural and functional networks

**Figure 3:**
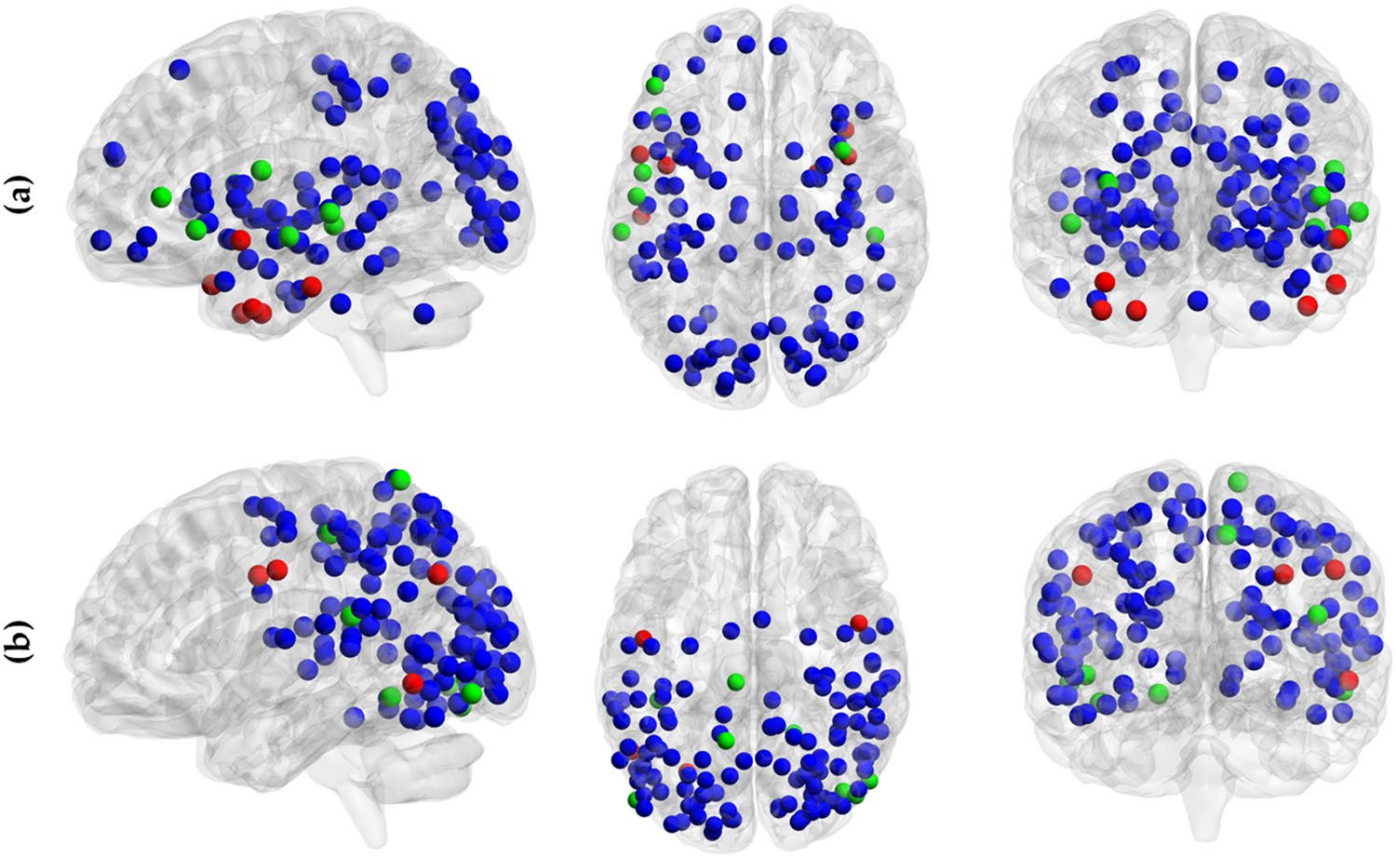
Rich club organization of (**a**) structural networks and (**b**) functional networks for cannabis users and healthy controls. The common rich club nodes in two groups are shown in blue. Few rich club nodes were only found for healthy controls (in red) or cannabis users (in green).

Figure 3 and Tables S9, S10 show the spatial distribution of structural and functional rich-club nodes for both groups. As shown, the structural rich club nodes were mostly distributed within left bilateral frontal, temporal and centro-occipital areas as well as in deep brain structures for both groups. Compared to controls, the structural networks in cannabis users showed higher and lower number of rich-club nodes within the superior and inferior temporal gyri, respectively.

The functional rich-club nodes were mostly located within parietal and posterior areas for both groups showing minor differences. Cannabis users showed slightly fewer and more rich-club nodes within the centro-temporal and parietal areas, respectively.

### Post hoc analysis

**Figure 4:**
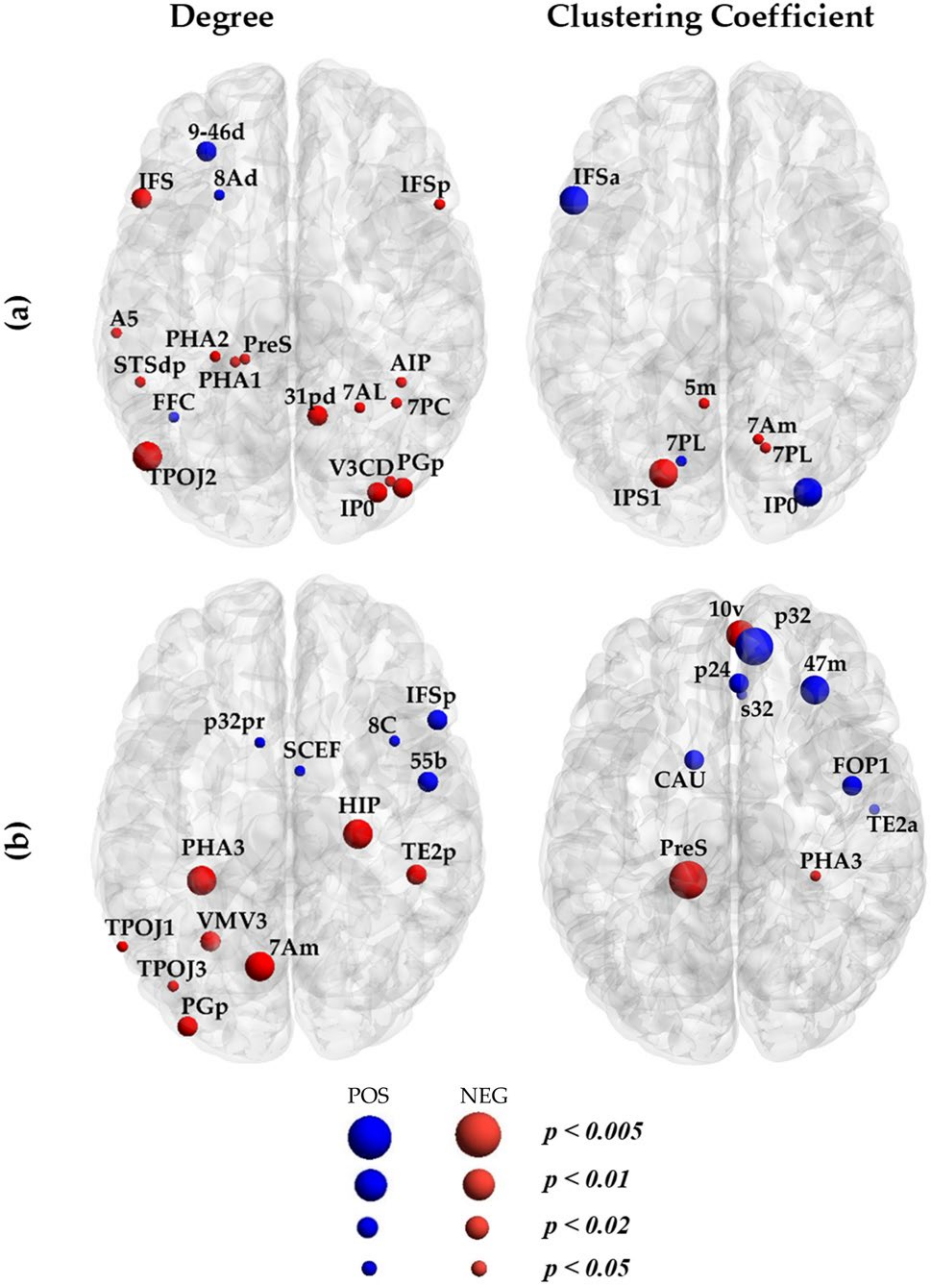
Regions showing significant association with times used cannabis in (**a**) structural and (**b**) functional networks. Nodes in red and blue show a negative (NEG) and positive (POS) association with times used cannabis, respectively. The node size represents the significant level (*p* < *0.05, p* < *0.02, p* < *0.01* and *p* < *0.005, uncorrected*) with larger nodes showing smaller p values. After FDR correction, only the PreS region was found to be statistically significant.

Figure 4 and Tables S5–S8 show the regression results illustrating regions whose graph measures were significantly (*p* < *0.05, uncorrected*) associated with TUC in structural (SN) and functional (FN) networks. In this figure, nodes represent a rate of change (β coefficients) in nodal degree and clustering coefficient higher than mean + 2SD with increasing TUC. Several regions in posterior areas showed a significant decrease in degree centrality with increasing TUC for structural networks (within the bilateral inferior frontal cortex, left temporo-parieto-occipital junction, right V3CD) and functional networks (within left parahippocampal area, left ventro-medial visual area, left superior parietal cortex, left inferior parietal cortex, right hippocampus, right medial temporal cortex). The degree of a few regions in SN (within left dorsolateral prefrontal cortex) and FN (within the right inferior frontal cortex, right premotor cortex) showed positive correlations with TUC. The clustering coefficient of several nodes within frontal and occipital areas was also positively correlated (*p* < *0.01, uncorrected*) with TUC for functional and structural networks, respectively. The left intra-parietal sulcus area in SN and left presubiculum and anterior cingulate and medial temporal cortices in FN were found to be negatively associated (*p* < *0.01, uncorrected*) with TUC. The left inferior frontal cortex and right intraparietal area in SN and right orbital and polar frontal cortex, right frontal opercular area, and left caudate in FN showed an inverse trend (Table S5). None of the aforementioned significant associations between network measures and TUC survived after FDR correction. Only significant association between degree and TUC within the presubiculum region survived after FDR correction.

## Discussion

Despite the high prevalence of cannabis use worldwide, little is known about its potential effect on the human brain. This study investigated the association of cannabis use with brain structural and functional connectivity using graph-theoretical analysis in a relatively large sample of cannabis users in comparison with healthy controls. Our results showed: (1) a small world topology and rich-club organization for brain structural and functional networks in cannabis users and healthy non-users, (2) no significant differences in global network measures between the groups, (3) significant decreases and increases in local integration and segregation of the structural/functional networks, respectively, in cannabis users compared to healthy controls, and (4) significant association between local measures of st ructural/functional networks and times used cannabis. Taken together, our findings showed altered regional properties of brain structural and functional networks in cannabis users.

Consistent with prior findings^9,12,16^, our results showed no significant alterations in global network properties of structural and functional brain networks in cannabis users in comparison with healthy controls (*p* > *0.05*, *uncorrected*). The small-world properties of both functional and structural networks were also found similar across the two groups, in line with previous studies^38^ on healthy individuals.

Our findings showed some regional alterations in structural networks associated with cannabis use especially in the cingulate^12^, dorsolateral^16^, frontal/posterior opercular, frontal medial cortex, insular and temporal regions. The structural connectivity alterations observed in these regions may be related to local changes in cortical gray matter thickness and the heterogeneous distribution of cannabinoid receptors across the brain associated with substance use disorder^32,39–42^. As more segregated networks tend to have high clustering coefficient^37^, the increase in clustering coefficient in some regions may suggest potential differences in the local processing capacity of these networks^12^. These different patterns of global and local metrics might reflect different sample characteristics in studies.

In the present data, we found rich-club nodes largely distributed in cortical and subcortical regions, in line with previous findings^19,23,34,43^. The structural rich club nodes were mainly found within bilateral frontal, temporal and centro-occipital areas and deep brain structures for both groups, whilst those of functional networks were mostly located within parietal and posterior areas. Compared to controls, our results revealed higher and lower numbers of rich-club nodes within the superior and inferior temporal gyri, respectively, for structural networks in cannabis users. This finding is in contrast with other studies^16^ reporting no differences in the rich-club organization of structural networks between cannabis users and healthy controls.

Our results showed that the functional rich-club nodes were mostly located within parietal and posterior areas for both groups displaying minor differences in number of rich-club nodes. Compared to controls, cannabis users showed slightly fewer and more rich-club nodes within centro-temporal and parietal areas, respectively. Few nodes within dorsal area showed a high level of rich-clubness for functional networks in cannabis users. This area has been reported to play an important role in habit formation in addictive behaviors^44^. These findings suggest potential aberrant connectome associated with cannabis use.

Our findings further showed significant associations between the nodal degree/clustering coefficient of structural/functional networks and the number of lifetime uses of cannabis. Consistent with previous findings^41^, clustering coefficients (measure of segregation) of structural connectivity showed positive associations with lifetime cannabis use mostly in the medial temporal cortex and negative associations in some regions including the dorsolateral prefrontal cortex. We also found a negative association between local parameters (degree of structural network, clustering coefficient of functional network) and TUC in the presubiculum region, located in the medial temporal cortex. The negative association between degree of functional network and times used cannabis was mostly found in the medial temporal cortex, the temporal-parietal-occipital junction and hippocampus. The hippocampus is one of the brain regions with the highest levels of expression of CB1 receptors^45^. The CB1-related structural and functional changes have been frequently found in this region in both human and animal models^16^. However, positive associations between the clustering coefficient of functional network and TUC were mostly observed in the anterior cingulate and medial prefrontal cortex. Some studies^16,21^ reported no significant association between the duration of cannabis use, times used cannabis, age of onset, and the detrimental effect on brain networks while others believe that an earlier onset or longer cannabis use may affect brain networks^15,46^. These inconsistencies may be due to differences in self-reported scores, cannabis user population across different studies and differences in methodology.

The current study has several limitations. First, the HCP database is a cross-sectional database which provides limited information about cannabis use and addiction. The existing measures like the age of onset are level-based and not exact. The alterations in connectivity patterns found in cannabis users might be due to patterns of daily use or chronicity. Second, in this cross-sectional database, only young adults aged 22–36 years were included. Longitudinal data are required to better characterize within-sample changes in connectivity patterns over time. Finally, rs-fMRI and functional connectivity are now widely considered to be dynamic over time, therefore a dynamic connectivity analysis might better illustrate time-varying patterns of connectivity associated with cannabis use.

## Conclusions

The present study investigated association between cannabis use and brain structural and functional connectivity. A graph-theoretic analysis was performed on whole-brain functional and structural networks of cannabis users and healthy controls in order to identify alterations in brain connectivity associated with cannabis use. Brain networks of both groups exhibited small-world properties. Furthermore, our findings suggested regional effects on network segregation and integration measures, being more significant in the insular, frontal opercular and lateral/medial temporal cortices. However, the global properties of the brain networks remain intact. The rich-club analysis of both structural and functional network indicated a typical pattern, although some minor differences were observed between the two groups. A negative association was observed between times used cannabis and regional structural and functional network measures in certain regions, including the hippocampus and presubiculum, which have been found in other studies to exhibit a high concentration of CB1-receptors. In future work, we will investigate time-varying changes in resting state functional connectivity patters in cannabis users.

## Supplementary Information

Supplementary Tables.

## Data Availability

The dataset analyzed in the current study is publicly available in the Human Connectome Project (HCP) repository on https://www.humanconnectome.org/study/hcp-young-adult/document/1200-subjects-data-release.
