# Supplementary material for: Altered brain structural and functional connectivity in cannabis users
Source: Sci Rep. 2023 Apr 10;13:5847. doi: 10.1038/s41598-023-32521-8 (PMC10086048; doi:10.1038/s41598-023-32521-8)
Supplement: Supplementary file 1 — Supplementary Tables. [file 41598_2023_32521_MOESM1_ESM.docx]

Table S1. Regions showing significant (*p<0.05*) differences between cannabis users and normal controls in the degree centrality of structural networks.

| Region | Area Name | Section | p-value | t-statistic |
| --- | --- | --- | --- | --- |
| OP1 | Area OP1/SII | Posterior opercular cortex | 0.02 | 2.22 |
| OP2~3_L_ | Area OP2~3/VS |  | 0.015 | -2.45 |
| POI1_R_ | Area Posterior Insular 1 | Insular and frontal opercular cortex | 0.007 | -2.72 |
| FOP5_L_ | Area Frontal Opercular 5 |  | 0.0007 | -3.45 |
| 33pr_R_ | Area 33 Prime | Anterior cingulate and medial prefrontal cortex | 0.02 | -2.34 |
| 23c_L_ | Area 23c | Paracentral lobular and mid-cingulate cortex | 0.013 | 2.49 |
| POS2_R_ | Parieto-Occipital Sulcus Area 2 | Posterior cingulate cortex | 0.006 | -2.77 |
| DVT_R_ | Dorsal Transitional Visual Area |  | 0.042 | -2.04 |
| 31a_L_ | Area 31a |  | 0.034 | 2.14 |
| PF_L_ | Area PF Complex | Inferior parietal cortex | 0.005 | -2.84 |
| V7_R_ | Seventh Visual Area | Dorsal stream visual cortex | 0.02 | 2.35 |
| V6_R_ | Sixth Visual Area |  | 0.001 | -3.26 |
| TGd_R_ | Area TG dorsal | Lateral temporal cortex | 0.009 | -2.61 |
| V3CD_L_ | Area V3CD | MT+ complex and neighboring areas | 0.002 | 3.04 |
| CAU_L_ | Caudate | SUBCORTICAL | 0.03 | 2.16 |

Table S2. Regions showing significant (*p<0.05*) differences between cannabis users and normal controls in the clustering coefficient of structural networks.

| Region | Area Name | Section | p-value | t-statistic |
| --- | --- | --- | --- | --- |
| 6r | Rostral Area 6 | Premotor cortex | 0.01 | 2.52 |
| i6~8_R_ | Inferior 6-8 Transitional Area | Dorsolateral prefrontal cortex | 0.042 | 2.05 |
| FOP4_R_ | Frontal Opercular Area 4 | Insular and frontal opercular cortex | 0.027 | 2.22 |
| POI1_R_ | Area Posterior Insular 1 |  | 0.017 | 2.40 |
| Ig_R_ | Insular Granular Complex |  | 0.027 | 2.23 |
| FOP5_L_ | Area Frontal Opercular 5 |  | 0.009 | 2.61 |
| 44_L_ | Area 44 | Inferior frontal cortex | 0.04 | 2.009 |
| IFJp_L_ | Area IFJp |  | 0.015 | 2.44 |
| IFSp_L_ | Area IFSp |  | 0.04 | 2.02 |
| IFJa_R_ | Area IFJa |  | 0.036 | 2.10 |
| OP2~3_L_ | Area OP2~3/VS | Posterior opercular cortex | 0.04 | 1.98 |
| PF_R_ | Area PF Complex | Inferior parietal cortex | 0.034 | 2.27 |
| VVC_R_ | Ventral Visual Complex | Ventral stream visual cortex | 0.019 | -2.35 |
| V3CD_L_ | Area V3CD | MT+ complex and neighboring areas | 0.016 | -2.42 |

Table S3. Regions showing significant (*p<0.05*) differences between cannabis users and normal controls in the degree centrality of functional networks.

| Region | Area Name | Section | p-value | t-statistic |
| --- | --- | --- | --- | --- |
| Ig_R_ | Insular Granular Complex | Insular and frontal opercular cortex | 0.03 | 2.20 |
| FOP3_L_ | Frontal Opercular Area 3 |  | 0.003 | -2.82 |
| A1_R_ | Primary auditory cortex | Early auditory cortex | 0.043 | 1.98 |
| RI_L_ | Retro Insular Cortex |  | 0.04 | 1.99 |
| TE1m_R_ | Area TE I Middle | Lateral temporal cortex | 0.041 | -1.92 |
| PGp_L_ | Area PGр | Inferior parietal cortex | 0.03 | -2.08 |
| V1_R_ | Primary visual cortex | Primary visual cortex | 0.04 | 1.88 |

Table S4. Regions showing significant (*p<0.05*) differences between cannabis users and normal controls in the clustering coefficient of functional networks.

| Region | Area Name | Section | p-value | t-statistic |
| --- | --- | --- | --- | --- |
| FFC | Fusiform Face Complex | Ventral stream visual cortex | 0.008 | 2.67 |
| FST | Area FST | MT+ complex and neighboring areas | 0.04 | 2.03 |
| 8Av_R_ | Area 8Av | Dorsolateral prefrontal cortex | 0.015 | -2.44 |
| p9-46v_L_ | Area posterior 9-46v |  | 0.03 | -2.27 |
| IFJa_L_ | Area IFja | Inferior frontal cortex | 0.007 | 2.69 |
| 6mp_L_ | Area 6mp | Paracentral lobular and mid-cingulate cortex | 0.03 | 2.18 |
| 33pr_L_ | Area 33 prime | Anterior cingulate and medial prefrontal cortex | 0.04 | -1.98 |
| FOP4_R_ | Frontal Opercular Area 4 | Insular and frontal opercular cortex | 0.03 | 2.15 |
| 52_R_ | Area 52 | Early auditory cortex | 0.03 | -2.14 |
| TGd | Area TG dorsal | Lateral/Medial temporal cortex | 0.002 | 2.24 |
| PHA2_R_ | Para Hippocampal Area 2 |  | 0.006 | -2.77 |
| TPOJ1_L_ | Area Temporo-Parieto-Occipital Junction 1 | Temporo-Parieto-Occipital junction | 0.04 | 2.05 |
| 7m_L_ | Area 7m | Posterior cingulate cortex | 0.02 | 2.14 |
| DV_R_ | Diencephalon Ventral | SUBCORTICAL | 0.009 | -2.62 |

Table S5. Regions whose degrees were significantly associated with times used cannabis (structural networks)

| Region | Area Name | Section | p-value | t-statistic |
| --- | --- | --- | --- | --- |
| 8Ad_L_ | Area 8Ad | Dorsolateral prefrontal cortex | 0.02 | 2.31 |
| 9~46d_L_ | Area 9~46d |  | 0.01 | 2.45 |
| IFSp_R_ | Area IFSp |  | 0.04 | -2.01 |
| PGp_R_ | Area PGp |  | 0.01 | -2.51 |
| IP0_R_ | Area Intraparietal 0 |  | 0.01 | -2.44 |
| IFSa_L_ | Area IFSa |  | 0.01 | -2.62 |
| A5_L_ | Auditory 5 Complex | Auditory association cortex | 0.02 | -2.26 |
| STSdp_L_ | Area STSd posterior |  | 0.03 | -2.14 |
| PHA1_L_ | Para Hippocampel Area 1 | Medial temporal cortex | 0.02 | -2.27 |
| PreS_L_ | PreSubiculum |  | 0.04 | -2.01 |
| PHA2_L_ | Para Hippocampal Area 2 |  | 0.03 | -2.17 |
| TPOJ2_L_ | Area Temporo-Parieto-Occipital junction 2 | Temporo-Parieto-Occipital junction | 0.006 | -2.79 |
| 7AL_R_ | Lateral Area 7A | Superior Partial cortex | 0.04 | -2.01 |
| 7PC_R_ | Area 7PC |  | 0.03 | -2.19 |
| AIP_R_ | Anterior Intraparietal Area |  | 0.03 | -2.15 |
| 31pd_R_ | Area 3 l pd | Posterior cingulate cortex | 0.01 | -2.39 |
| FFC_L_ | Fusiform Face Complex | Ventral stream visual cortex | 0.02 | 2.32 |
| V3CD_R_ | Area V3CD | MT+ complex and neighboring areas | 0.03 | -2.14 |

Table S6. Regions whose clustering coefficients were significantly associated with times used cannabis (structural networks)

| Region | Area Name | Section | p-value | t-statistic |
| --- | --- | --- | --- | --- |
| IFSa_L_ | Area IFSa | Inferior frontal cortex | 0.01 | 2.50 |
| 5m_L_ | Area 5m | Paracentral lobular and mid-cingulate cortex | 0.03 | -2.20 |
| 7PL | Lateral Area 7P | Superior/ Inferior parietal cortex | 0.03 | 2.10 |
| 7Am_R_ | Medial Area 7A |  | 0.02 | -2.36 |
| IP0_R_ | Area Intra Parietal 0 |  | 0.01 | 2.59 |
| IPS1_R_ | Intra Parietal Sulcus Area 1 | Dorsal stream visual cortex | 0.01 | -2.59 |
| V3B_L_ | Area V3B |  | 0.04 | -2.02 |

Table S7. Regions whose degrees were significantly (*p<0.05*) associated with times used cannabis (functional networks)

| Region | Area Name | Section | p-value | t-statistic |
| --- | --- | --- | --- | --- |
| PHA3 | Para Hippocampal Area 3 | Medial temporal cortex | 0.007 | -2.73 |
| TE2p_R_ | Area TE2 posterior |  | 0.015 | -2.48 |
| TPOJ1_L_ | Area Temporo-Parieto-Occipital Junction 1 | Temporo-Parieto-Occipital junction | 0.04 | -2.03 |
| TPOJ3_L_ | Area Temporo-Parieto-Occipital Junction 3 |  | 0.02 | -2.23 |
| 55b_R_ | Area 55b | Premotor cortex | 0.017 | 2.44 |
| SCEF_R_ | Supplementary and Cingulate Eye Field | Paracentral lobular and mid-cingulate cortex | 0.02 | 2.23 |
| 8C_R_ | Area 8C | Dorsolateral prefrontal cortex | 0.02 | 2.31 |
| IFSp_R_ | Area IFSP | Inferior frontal cortex | 0.013 | 2.54 |
| PGp_R_ | Area PGp | Inferior parietal cortex | 0.02 | -2.25 |
| 7Am_L_ | Medial Area 7A | Superior Parietal cortex | 0.008 | -2.71 |
| p32pr_L_ | Area p32 prime | Anterior cingulate and medial prefrontal cortex | 0.03 | 2.16 |
| VMV3_L_ | Ventro Medial Visual Area 3 | Ventral stream visual cortex | 0.01 | -2.63 |
| HIP_R_ | Hippocampus | SUBCORTICAL | 0.008 | -2.72 |

Table S8. Regions whose clustering coefficients were significantly (*p<0.05*) associated with times used cannabis (functional networks)

| Region | Area Name | Section | p-value | t-statistic |
| --- | --- | --- | --- | --- |
| 47m_R_ | Area 47m | Orbital and polar frontal cortex | 0.006 | 2.80 |
| 10v_R_ | Area 10v | Anterior cingulate and medial prefrontal cortex | 0.009 | -2.66 |
| FOP1_R_ | Frontal Opercular Area 1 | Posterior opercular cortex | 0.02 | 2.46 |
| PHA3_R_ | Para Hippocampal Area 3 | Medial temporal cortex | 0.04 | -2.01 |
| TE2a_R_ | Area TE2 anterior | Lateral temporal cortex | 0.03 | 2.13 |
| s32_R_ | Area s32 | Anterior cingulate and medial prefrontal cortex | 0.024 | 2.29 |
| P24_R_ | Area posterior 24 |  | 0.01 | 2.46 |
| P32_R_ | Area p32 |  | 0.003 | 3.06 |
| PreS_L_ | PreSubiculum | Medial temporal cortex | 0.003 | -3.05 |
| CAU_L_ | Caudate | SUBCORTICAL | 0.01 | 1.01 |

Table S9. Significant differences between two groups regarding the rich club organization of structural network

|  | Region | Area Name | Section |
| --- | --- | --- | --- |
| RC nodes in cannabis users | FOP4 | Frontal Opercular Area 4 | Insular and frontal opercular cortex |
|  | 6r | Rostral Area 6 | Premotor cortex |
|  | STSdp | Area STSd posterior | Auditory association cortex |
|  | A5 | Auditory 5 Complex |  |
|  | STSda | Area STSd anterior |  |
|  | 47l | Area 47l (47 lateral) | Inferior frontal cortex |
|  | P47r | Area posterior 47r |  |
| RC nodes in HC | PeEc | Perirhinal Ectorhinal Cortex | Medial temporal cortex |
|  | TGd | Area TG dorsal | Lateral temporal cortex |
|  | TGv | Area TG Ventral |  |
|  | TE2a | Area TE2 anterior |  |
|  | STGa | Area STGa | Auditory association cortex |

Table S10. Significant differences between two groups regarding the rich club organization of functional network

|  | Region | Area Name | Section |
| --- | --- | --- | --- |
| RC nodes  (Cannabis users) | LO1 | Area Lateral Occipital 1 | MT+ complex and neighboring areas |
|  | PIT | Posterior Infero-Temporal Complex | Ventral stream visual cortex |
|  | VMV1 | Ventro-Medial Visual Area 1 |  |
|  | V4t | Area V4t | MT+ complex and neighboring areas |
|  | 24d | Dorsal Area 24d | Paracentral lobular and mid-cingulate cortex |
|  | 5L | Area 5L |  |
|  | OP1 | Area OP1/SII | Posterior opercular cortex |
|  |  |  |  |
| RC nodes  (Healthy controls) | PEF | Premotor Eye Field | Premotor cortex |
|  | LIPd | Area Lateral Intra-Parietal dorsal | Superior Parietal cortex |
|  | PHT | Area PHT | Lateral temporal cortex |
